# Childhood mortality from acute diarrheal disease in Paraguay and vaccination impact: a 31-year ecological study

**DOI:** 10.4178/epih.e2026010

**Published:** 2026-02-20

**Authors:** María Eugenia Galeano, Magaly Martínez, Anibal Francisco Espínola, Eliana Andrea Alvarenga, Margarita Samudio

**Affiliations:** 1 Instituto de Investigaciones en Ciencias de la Salud (IICS), Universidad Nacional de Asunción (UNA), San Lorenzo, Paraguay; 2 Universidad del Pacífico, Dirección de Investigación, Asunción, Paraguay

**Keywords:** Diarrhea, Rotavirus, Vaccines, Child, Mortality, Paraguay

## Abstract

**OBJECTIVES:**

Acute diarrheal disease (ADD) remains a leading cause of morbidity and mortality in children under 5 worldwide. In Paraguay, the introduction of the rotavirus vaccine in 2010 aimed to reduce the burden of ADD in infants and young children. This study analyzed 31 years of public data on ADD morbidity and mortality in Paraguayan children under 5 and evaluated the impact of rotavirus vaccination.

**METHODS:**

Annual ADD incidence, total deaths, and mortality rates from 1993 to 2023 were obtained from public databases. Proportionate mortality due to ADD, cause-specific mortality rates, absolute risk reduction (ARR) and relative risk reduction (RRR), and joinpoint regression were calculated using EPIDAT 4.2 and Joinpoint 5.3.0 software, with data stratified by age group and vaccination period.

**RESULTS:**

Between 1993 and 2021, pediatric mortality in Paraguay declined steadily across all age groups. Following the introduction of Rotarix in 2010, infant mortality dropped from 2.36 to 0.19 deaths per 1,000 live births (ARR, 0.83; RRR, 81.4%). Joinpoint analysis showed a decline in under-5 mortality between 2009 and 2012 (annual percentage change=−35.2%, p<0.05), followed by stabilization. ADD incidence increased until 2019 and then declined, while vaccine coverage exceeded 70%.

**CONCLUSIONS:**

Paraguay has sustained declines in infant and childhood mortality over 3 decades through improvements in healthcare and immunization, with the 2010 introduction of Rotarix reducing the mortality rate by 43 deaths per 100,000 infants under 12 months. These results underscore the impact of vaccination and health system strengthening, while highlighting the need to monitor emerging threats.

## GRAPHICAL ABSTRACT

[Fig f5-epih-48-e2026010]

## Key Message

Despite the introduction of rotavirus vaccination in Paraguay, long-term trends in acute diarrheal disease remained understudied. This 31-year analysis reveals a substantial decline in ADD-specific mortality following vaccine implementation, especially in children aged 1–4. However, ADD incidence resurged after 2013, highlighting gaps in disease control beyond vaccination. These findings underscore the vaccine’s impact while exposing the need for integrated surveillance and broader public health strategies to sustain progress and address residual disease burden.

## INTRODUCTION

Acute diarrheal disease (ADD), also known as acute gastroenteritis, remains one of the leading causes of mortality among children under 5, particularly in low-income and middle-income countries [1]. In Paraguay, ADD continues to represent a substantial public health burden and remains among the leading infectious causes of child mortality, according to the Ministry of Public Health and Social Welfare (MSPyBS) [2]. Despite significant improvements in child health indicators since the 1990s, inequalities in access to sanitation, clean water, and healthcare persist, contributing to sustained transmission of enteric infections [3,4]. Rotavirus, a major cause of gastroenteritis and diarrhea, can lead to rapid dehydration and death in this age group; however, it is preventable through vaccination [5].

Rotavirus circulates widely in Paraguay, with multiple genotypes coexisting or alternating over time in both pediatric and adult populations [6-9]. Among enteric viruses, rotavirus and norovirus are the most frequently detected pathogens in Paraguayan children and are transmitted primarily via the fecal–oral route. The epidemiology and seasonal distribution of rotavirus infections in Paraguay have been documented since 2002 [7,8], while norovirus infections have been studied since at least 2013 [10] across different periods and in environmental samples [11].

Immunization remains the most effective preventive strategy against these infections. Although 2 norovirus vaccine prototypes are currently under development, neither has been approved by the Food and Drug Administration. In contrast, licensed rotavirus vaccines have been available since 2006 and have demonstrated efficacy [12].

Rotarix (GlaxoSmithKline, Philadelphia, PA, USA), a monovalent vaccine targeting the G1P [8] genotype, was incorporated into Paraguay’s National Immunization Program (Spanish: Programa Ampliado de Inmunizaciones [PAI]) of the MSPyBS in 2010. Administered orally at 2 months and 4 months of age, the vaccine achieved coverage exceeding 60% by the following year and over 70% in subsequent years [13,14]. Global and regional evidence demonstrates that rotavirus vaccination markedly reduces severe diarrhea, hospitalizations, and mortality in children under 5 [12,15-17].

However, few studies have evaluated the national impact of rotavirus vaccination in Paraguay, particularly across the pre-vaccination and post-vaccination eras. Understanding these trends is essential to guide policies aimed at further reducing childhood mortality and to address the emerging predominance of norovirus infection.

This study aimed to analyze 31 years of national data on the incidence, morbidity, and mortality of ADD in Paraguayan children under 5 and to evaluate the impact of the 2010 introduction of Rotarix within the National Immunization Program.

## MATERIALS AND METHODS

### Study design and population

We conducted an ecological, retrospective, analytical study using a quantitative approach. Acute diarrhea was defined as the onset of 3 or more loose or watery stools per day lasting 14 days or less [18]. The study focused on reported ADD cases and the number and rates of deaths due to ADD among infants, children aged 1 year to 4 years, and children under 5 years in Paraguay, considering time as the primary variable (epidemiological year and the pre- and post-rotavirus vaccination periods).

### Data collection

Data from 1993 to 2023 were retrieved. ADD case data (2011, 2013–2023) were obtained from the Compulsory Notifiable Disease Surveillance System (ENO) of the Directorate General of Health Surveillance (DGVS), MSPyBS [19], and from weekly epidemiological reports [2]. Mortality data (1997–2021), by age group and epidemiological region, were extracted from the Historical Mortality Indicator Series (INDIMOR) [20], which is compiled using the vital statistics information subsystem (live births and deaths) as the primary source. Vaccination coverage among children under 5 years of age (2010–2023) was obtained from PAI bulletins [13]. All datasets were accessed under the public information license of the Paraguayan government [21]. Additional data on population counts and the under-5 mortality rate were obtained from international organizations [22,23], as detailed in Supplementary Material 1. Raw data can be requested directly from the first author.

### Data management and statistical analysis

Spreadsheets, tables, and graphs were created using Excel for Mac version 16.77 (Microsoft Corp., Redmond, WA, USA). Descriptive statistics were used to summarize quantitative variables as absolute frequencies and relative percentages, with 95% confidence intervals (CIs). Means and standard deviations were calculated and stratified by age group and time period for each variable. Statistical analyses were performed using EPIDAT version 4.2 (Xunta de Galicia; PAHO/WHO) [24].

Three periods were defined: (1) from the earliest available data through 1999, when Paraguay implemented health service reforms [25]; (2) the pre-vaccination period, from 2000 to 2009; and (3) the post-vaccination period, from 2010 through the last year with available data. We calculated the mean mortality rate for each population and period using INDIMOR data (Supplementary Materials 2-4) by dividing the sum of annual mortality rates by the number of years included in the respective period.

Variance tests (*F*-tests) and Student *t*-tests for independent samples were used to assess differences in mean mortality rates across the 3 periods.

Proportionate mortality (PM), defined as the proportion of ADD-related deaths among all deaths within each pediatric population across the 3 study periods, was calculated for each age group according to the Pan American Health Organization definition [26]:


$$PM=\frac{Number\;of\;deaths\;due\;to\;ADD}{Total\;number\;of\;deaths}\times 100$$

Results were expressed as percentages.

The cause-specific mortality rate (CSMR), a measure of the frequency of deaths attributed to a specific cause (i.e., ADD) within a defined population and time period, was calculated to better quantify the burden of ADD-related mortality using the following formula:


$$CSMR=\frac{Number\;of\;deaths\;due\;to\;ADD}{Total\;population\;at\;risk}\times 100,000$$

The denominator was the age-specific population corresponding to each subgroup analyzed. Results were expressed as deaths per 100,000 children at risk (Supplementary Materials 2-4).

To evaluate differences in PM between periods (1 vs. 2, 1 vs. 3, and 2 vs. 3), we calculated the differences in PM, along with Z-scores and p-values (Supplementary Material 5). Supplementary Material 6 summarizes mean annual CSMRs and the differences between periods. All statistical tests used a significance level of 0.05 (95% CI).

Incidence was estimated as the annual number of reported ADD cases per 1,000 children under 5, using DGVS population data (2011 and 2013–2023) (Supplementary Material 7). Absolute risk reduction (ARR), defined as the difference in mortality rates between the pre-vaccination and post-vaccination periods, was calculated to quantify the decline in ADD-related mortality attributable to the introduction of Rotarix within the PAI, according to standard epidemiological methods [27]:


$$ARR={\bar{\mu }}_{pre}-{\bar{\mu }}_{post}$$

where ${\bar{\mu }}_{pre}$ and $\bar{{\bar{\mu }}_{post}}$ represent the mean mortality rates during the pre-vaccination period (1999–2009) and the post-vaccination period (2010–2021), respectively.

Relative risk reduction (RRR) in ADD mortality was calculated as:


$$RRR=\frac{ARR}{{\bar{\mu }}_{pre}}$$

where RRR is the ratio of ARR to the mean mortality rate during the pre-vaccination period, ${\bar{\mu }}_{pre}$, expressed as a percentage.

#### Trend analysis using Joinpoint regression model

Trend analysis was performed using the Joinpoint Regression Program version 5.3.0 (National Cancer Institute, Rockville, MD, USA). We applied a segmented Poisson log-linear model (ln(y)=βx+e) to estimate the annual percentage change (APC) and average annual percentage change (AAPC), with year as the independent variable and pediatric mortality rate as the dependent variable. Joinpoints indicating significant shifts in trends were selected based on the Bayesian information criterion. Up to 3 joinpoints per regression were tested, assuming homoscedastic, uncorrelated errors, with statistical significance set at p<0.05.

### Ethics statement

The study was approved by the Institutional Ethics Committee of the Instituto de Investigaciones en Ciencias de la Salud, Universidad Nacional de Asunción (CEI-IICS No. 26/2024). All population-based data were anonymized, handled confidentially, and stored securely by the principal investigator. Data will be shared with other research groups in accordance with open science principles and authorship rights.

## RESULTS

### Characteristics of the data

The analysis included data on total counts and mortality rates in infants, children aged 1 year to 4 years, and children under 5 years in Paraguay, including all-cause mortality and ADD-specific mortality, spanning 29 years (1993–2021) (Supplementary Materials 1-4). We also analyzed ADD incidence among pediatric cases for 12 years (2011 and 2013–2023) and rotavirus vaccination coverage for 14 years (2010–2023) (Supplementary Material 7).

### Mortality from acute diarrheal disease in Paraguay

In Paraguay, both all-cause mortality and ADD-related mortality showed a consistent downward trend across all age groups from 1993 to 2021 (Supplementary Materials 2-4). For comparisons of mortality rates and linear regression trends between the pre-vaccination and post-vaccination periods, data from 2000 to 2021 were selected and are presented in Table 1.

**Figure 1.**
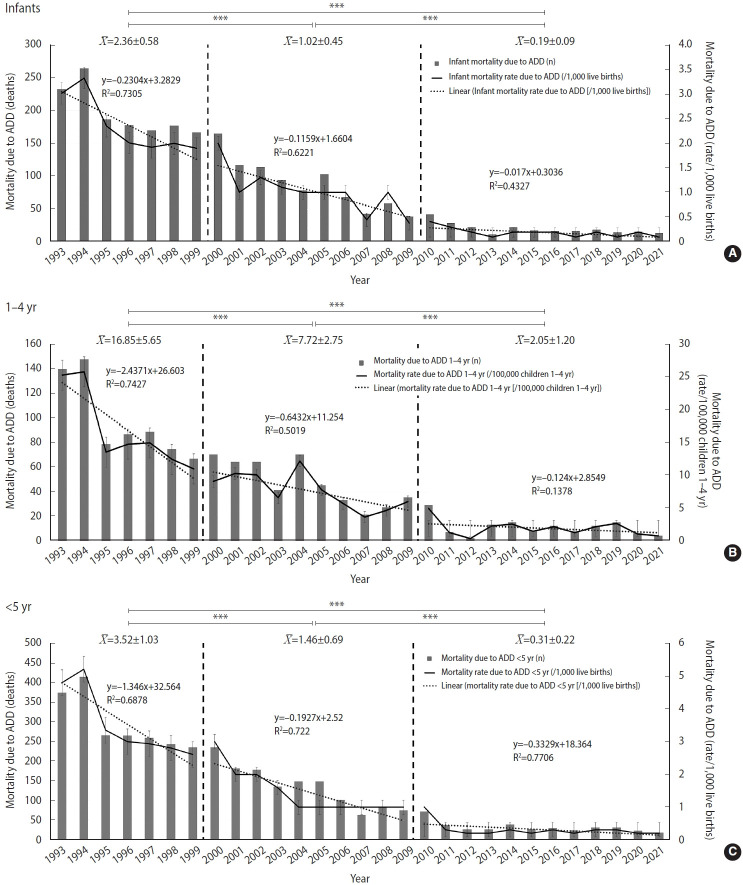
Pediatric mortality distribution and mortality rates due to acute diarrheal disease (ADD) among children under 5 years in Paraguay, stratified by age group. (A) Infants (up to 12 months of age). (B) Children aged 1 to 4 years. (C) Children under 5 years. The solid line indicates mortality rates (secondary y-axis). Dashed vertical lines mark period boundaries: period 1 (1993–1999), period 2 (2000–2009; pre-vaccination), and period 3 (2010-2021; post-vaccination). $\bar{X}$ represents the mean mortality rates for periods 2 and 3. ***p<0.001 for all comparisons; 95% confidence interval.

Figure 1 shows the distribution of pediatric ADD mortality and mortality rates among children under 5 years in Paraguay, stratified by age group; mean mortality rates are also provided. Statistically significant differences are indicated (p<0.001 for all comparisons; 95% CI).

### Acute diarrheal disease morbidity in the pediatric population

**Figure 2.**
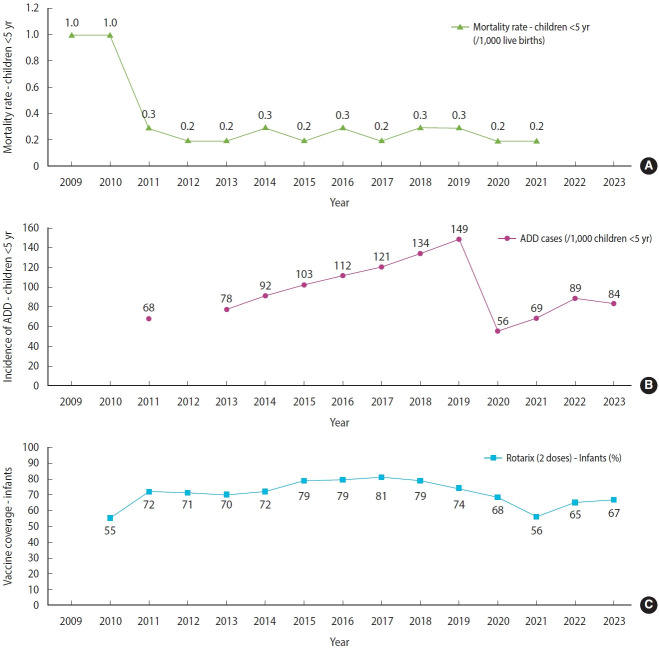
(A) ADD mortality rate among children under 5 years. (B) ADD incidence. (C) Proportion of infants (<1 year) with complete rotavirus vaccination (Rotarix, 2 doses) in Paraguay from 2009 to 2023. ADD, acute diarrheal disease.

Annual trends in ADD incidence, rotavirus vaccination coverage, and under-5 mortality from 2009 to 2023 are presented in Supplementary Material 7. ADD incidence increased progressively through 2019, followed by a sharp decline and subsequent stabilization. Mortality rates among children under 5 declined markedly after 2010 and remained consistently low throughout the study period. Complete vaccination coverage (Rotarix, 2 doses) in infants ranged from 55% to 81% (mean, 76%; 95% CI) for most of the period, but declined after 2018 (Figure 2A-C).

### Pediatric mortality trends and vaccine-associated risk reduction

**Figure 3.**
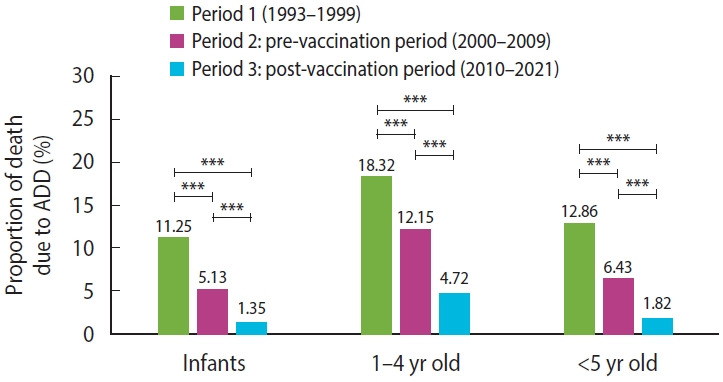
Proportionate mortality due to acute diarrheal disease (ADD) among Paraguayan children by age group and period. ***p<0.001 for all comparisons; 95% confidence interval.

Supplementary Materials 2-4 summarize PM, CSMR, and the ranking of ADD among infectious causes of death in Paraguayan children. PM declined significantly from the pre-vaccination to the post-vaccination period among children aged 1–4 years (12.15%; 95% CI, 7.36 to 25.39 vs. 4.72%; 95% CI, 1.22 to 9.93; p=0.002), with age-dependent differences in the magnitude of effect (Supplementary Material 5). Overall, reductions in PM were significant across all age groups (p<0.001 for all comparisons; 95% CI) (Figure 3). CSMR also decreased consistently, most notably among infants, with a decline of 110 deaths per 100,000 from 1993 to 2021 (Supplementary Material 6).

ARR and RRR in ADD-related mortality among children under 5 are summarized in Table 2. Mortality declined across all age groups, with the most pronounced reduction occurring after rotavirus vaccine introduction. Among infants, mortality decreased from 2.36 to 0.19 deaths per 1,000 live births between 1993–1999 and the post-vaccination period, corresponding to an ARR of 0.83 and an RRR of 81.4%. Similar downward trends were observed among children aged 1-4 years and in the overall under-5 group.

### Joinpoint regression analysis

**Figure 4.**
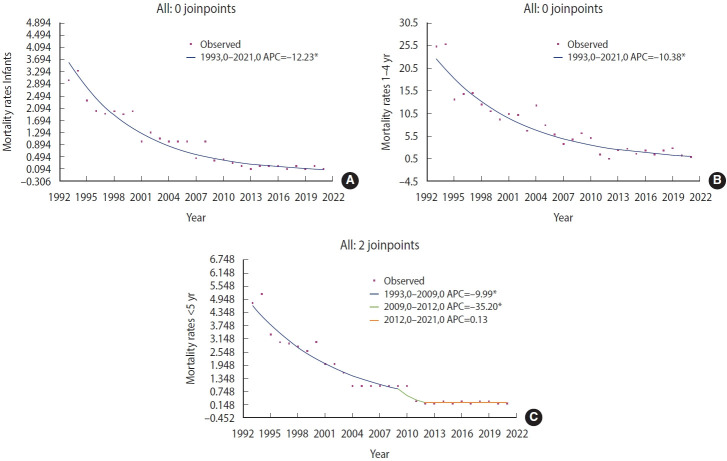
Trend analysis using the Joinpoint regression model based on a log-linear model (ln(y)=βx+e), with year (1992–2022) as the independent variable and pediatric acute diarrheal disease mortality rates as the dependent variable. (A) Infants (up to 12 months). (B) Children aged 1 to 4 years. (C) Children under 5 years. Annual percent change (APC) is shown in the inset box. *p<0.05.

Joinpoint regression revealed distinct temporal patterns in mortality across age groups (Figure 4A-C, Supplementary Material 8A-C). Among children under 5 and infants, 2 significant joinpoints were detected: mortality declined moderately until 2009–2010, followed by a sharp reduction between 2010 and 2012 (APC=–35.20%, p<0.05), and then stabilized.

In contrast, mortality among children aged 1–4 years showed a steady, continuous decline throughout the study period, with no joinpoints detected, indicating sustained improvement in post-infancy survival (Supplementary Material 8A-C).

## DISCUSSION

This study provides the first long-term analysis of trends in childhood mortality in Paraguay over nearly 3 decades, together with 12 years of data on ADD incidence in pediatric cases and 14 years of rotavirus vaccination coverage in infants. We show how patterns of ADD-related mortality in children have changed over time. ADD was consistently the second leading infectious cause of mortality among children aged 1 year to 4 years and the third among infants and children under 5 in Paraguay for several years (Supplementary Materials 3 and 4), before ranking third overall in 2021. This shift may reflect multiple factors discussed below, including an increased burden of respiratory infections and coronavirus disease 2019 (COVID-19), as well as a rise in meningitis cases [20].

### Mortality patterns

Our results show that pediatric mortality rates in Paraguay, both all-cause and ADD-specific, have declined steadily across all age groups since 1993, with the most pronounced reductions observed among infants after 2010 (Supplementary Materials 2-4). Linear regression analysis supported these findings: the mean infant mortality rate decreased significantly from 1.02±0.45 to 0.19±0.09 deaths per 1,000 live births, with a strong temporal association, particularly during the transition to the post-vaccination period. A similar sustained downward trend, followed by a smaller post-vaccination regression slope, was observed among children under 5 years of age. Among children aged 1-4 years, the mortality rate showed a moderate linear decline that slowed after vaccination, likely because rates had reached very low levels (Figure 1 and Table 1).

Historically, Paraguay has faced substantial health inequalities, with pronounced geographic disparities in child mortality and malnutrition. In the 1990s, under-5 mortality ranged from 17.4 per 1,000 live births in the capital, Asunción, to over 50 per 1,000 in remote departments (e.g., Boquerón and Alto Paraguay), while chronic malnutrition affected 3.7% to 14.6% of children and contributed to 13% of deaths [3,4]. Limited access to safe water and sanitation, particularly in rural areas, further amplified the burden of communicable diseases, with diarrheal illnesses emerging as a major cause of pediatric mortality [4]. By 2000, ADD accounted for 3 deaths per 1,000 live births among children under 5 [20].

The temporal dynamics revealed by Joinpoint regression highlight age-specific differences in the pace and timing of mortality reduction in Paraguay. An accelerated decline followed by stabilization after 2012 suggests a plateau in mortality reduction. Specifically, in the under-5 population, 2 joinpoints were identified in 2009 and 2012. The APC shifted significantly from –9.99% (1993–2009, p<0.05) to –35.20% (2009–2012, p<0.05), followed by stabilization after 2012, with an APC of 0.13%, which was not statistically significant (p=0.76; Figure 4C).

Although the incidence of ADD increased through 2019, mortality rates continued to decline after 2011. Vaccination coverage remained above 70% for most of the study period but decreased after 2018. This divergence suggests that rotavirus vaccination primarily reduced the severity and fatality of diarrheal disease rather than its occurrence, consistent with global evidence [12,15,17]. Continued circulation of other enteric pathogens—particularly norovirus—may also explain the persistent burden of ADD despite declining deaths [6,10]. Therefore, the post-vaccination trends observed in Paraguay likely reflect both mechanisms.

Rotarix is a monovalent live-attenuated rotavirus vaccine targeting the G1P [8] genotype, which has historically been one of the most prevalent genotypes worldwide, including in North America, Europe, and Asia [12]. The vaccine has demonstrated more than 90% efficacy in preventing severe diarrhea and hospitalization [17].

Rotavirus remains a leading cause of diarrhea requiring hospitalization among children under 5 worldwide. In South America, however, norovirus has become a predominant cause, accounting for 22.2% (95% CI, 17.5 to 27.9) of diarrheal cases in the region [5]. The widespread use of rotavirus vaccines likely contributed to this shift. Globally, 120 countries have introduced rotavirus vaccination, achieving a final-dose coverage of 51%, although coverage varies substantially by region [28]. In Paraguay, coverage has been consistently higher—67% in 2023 and an average of 72% since 2010, approximately 21 percentage points above the global mean (Supplementary Material 7). This sustained uptake reflects strong program implementation and public acceptance and likely contributed to greater declines in rotavirus-related morbidity and mortality.

Regional evidence supports this interpretation. The early introduction of rotavirus vaccination in Brazil, El Salvador, Mexico, Nicaragua, and Panama in 2006 led to significant reductions in diarrhea-related deaths compared with countries that adopted the vaccine later, including Paraguay. Between 2002 and 2009, an estimated 1,777 deaths per year among children under 5 were averted in the early-adopting countries [15]. These results underscore the major impact of rotavirus vaccination on child survival while highlighting the emerging need to address the growing burden of norovirus infection through new preventive strategies.

In September 1990, the World Summit for Children established targets for the year 2000, along with intermediate goals for 1995. Three quantitative objectives focused on education, nutrition, and environmental health, including a 20% reduction in the prevalence of protein–energy malnutrition in children under 5 and reductions in the proportion of the population lacking access to drinking water and basic sanitation; these efforts were intended to promote childhood survival, development, and protection [16]. In Paraguay, the MSPyBS undertook a health sector reorganization by 1996 to align with the Summit’s goals. This reform prioritized prevention, integration of services for low-income populations, and decentralization, while also addressing key health determinants such as sanitation, food security, and infrastructure [25]. No other major government decisions related to public health improvement were made during this period. These efforts likely contributed to the decline in under-5 mortality observed from 1993 to 1999, as documented in this study.

Previous studies have documented trends in diarrhea-related mortality in Paraguay [29,30]. The reductions in pediatric mortality since 1999 likely reflect a combination of factors, including improved detection and management of viral and bacterial infections, broader availability of molecular diagnostics, improved healthcare infrastructure, more rational antibiotic use, and increased public health awareness, alongside overall advances in medical care [31].

ADD incidence increased sharply from 2013 to 2019, followed by a marked decline in 2020 attributable to the COVID-19 pandemic and associated quarantine measures, with a subsequent increase to 84.7 per 1,000 inhabitants in 2023 (Figure 2). Shioda et al. [32] reported that, in addition to declining mortality in the post-vaccination period, hospitalizations in Paraguay due to rotavirus gastroenteritis decreased, with a 6.5% relative reduction for every 10.0% increase in vaccination coverage. The seasonal hospitalization peaks also changed after vaccine introduction, becoming smaller and occurring later in the season [32].

### Mortality risks

Overall, the sustained decline in absolute and relative mortality risk underscores the long-term impact of public health interventions and broader improvements in child health outcomes. In children under 5, the ARR between the pre-vaccination and post-vaccination periods was 1.15±0.74 deaths per 1,000 live births and was statistically significant (t=3.436; 95% CI; p<0.05), with a significant RRR of 78.8% (Table 2). In contrast, although the reduction in mortality among infants after vaccine introduction was substantial, the ARR was 0.83±1.57 deaths per 1,000 live births and did not reach statistical significance (t=1.129; 95% CI; p=0.277), whereas the RRR (81.4%) did. This apparent discrepancy reflects differences in how these measures quantify effect. ARR is based on the absolute difference in rates, which may be small when baseline mortality is already low; consequently, even a large proportional reduction may translate into a modest absolute change, limiting statistical power. By contrast, RRR expresses the proportional decline relative to baseline risk, thereby emphasizing the magnitude of effect regardless of the underlying event rate. Thus, the statistical significance of the RRR reflects a robust proportional reduction, whereas the non-significant ARR likely reflects limited precision due to the small number of events in this age group.

The PM due to ADD among children aged 1 year to 4 years declined significantly after 2010 (p=0.002; 95% CI). PM during the pre-vaccination period (2000–2009) was 12.15%, decreasing to 4.72% in the post-vaccination period (2010–2021). This decline may also have contributed to the change in ADD’s rank among infectious causes of pediatric mortality. Finally, the CSMR results suggest that deaths were averted through the combined implementation of public health and sanitation measures across all age groups, with the greatest impact observed among infants. Specifically, CSMR decreased by 43 deaths per 100,000 infants following the introduction of Rotarix. This impact is comparable to the combined effect of interventions implemented to reduce ADD mortality between 1993 and 1999, underscoring the pivotal role of rotavirus vaccination in sustaining long-term improvements in child survival.

### Limitations

A limitation of this study is the incompleteness of publicly available data for certain years, which prevented the inclusion of some data points—particularly the number of ADD cases before rotavirus vaccine implementation. In addition, data on ADD etiology were not accessible; therefore, morbidity and mortality could not be categorized by viral agent. This limitation may bias the results, which should therefore be interpreted with caution. We also acknowledge that information on key determinants that could influence ADD morbidity and mortality was unavailable and therefore not analyzed, including hospitalization records, healthcare access, diagnostic improvements over time, children’s nutritional status, and ADD severity. These factors are important for evaluating pediatric mortality at the individual level.

In conclusion, over 3 decades, Paraguay has achieved a sustained decline in infant and childhood mortality, largely driven by improvements in sanitation, healthcare access, and immunization coverage. The introduction of the rotavirus vaccine in 2010 marked a critical inflection point, consolidating earlier gains achieved through public health interventions during the 1990s. These findings highlight the cumulative and synergistic effects of vaccination and broader health system strengthening on child survival. Continued efforts to maintain high vaccine coverage and address residual inequities will be essential to further reduce preventable deaths and to confront the emerging predominance of norovirus as a leading cause of severe childhood diarrhea in the post-rotavirus vaccination era.

## Figures and Tables

**Figure f5-epih-48-e2026010:**
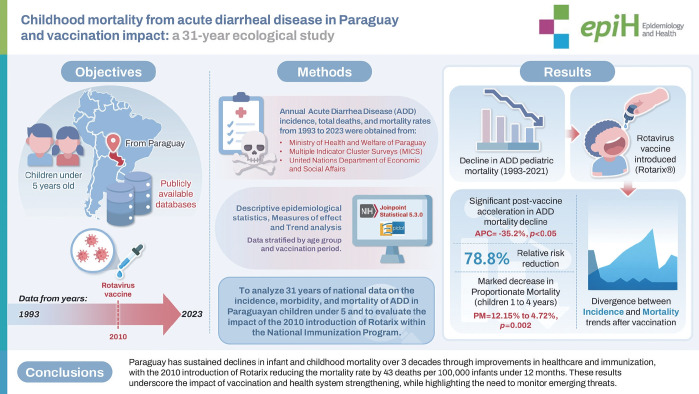


**Table 1.** Mean mortality rate and linear regression results for the pre- and post-vaccination periods (95% CI; p<0.05)

Age (yr)	Mean mortality rate per 1,000		Correlation coefficient		*t*-value^1^	p-value^2^
	Pre-vaccination period (2000–2009)	Post-vaccination period (2010–2021)	Pre-vaccination period	Post-vaccination period		
Infants (live births)	1.02±0.45	0.19±0.09	0.79	0.66	5.79	<0.05
1–4 (children 1–4 yr old)	0.08±0.03	0.02±0.01	0.71	0.37	6.05	<0.05
≤5 (live births)	1.46±0.69	0.31±0.22	0.85	0.88	5.06	<0.05

1 Student *t*-statistic.

2 Probability that the observed association occurred by chance.

**Table 2.** Mortality rates and risk reduction metrics by age group and period

Age group	Mortality rate due to ADD (mean)^1^			ARR^2^		Relative risk reduction (ARR, %)^3^	
	Period 1 (1993–1999)	Period 2: Pre-vaccination (2000–2009)	Period 3: Post-vaccination (2010–2021)	Period 1 vs. 2	Period 2 vs. 3	Period 1 vs. 2	Period 2 vs. 3
Infants	2.36	1.02	0.19	1.34	0.83	56.7	81.4
Children 1–4 yr old	0.17	0.08	0.02	0.09	0.06	53.0	73.4
Children <5 yr old	3.52	1.46	0.31	2.06	1.15	58.5	78.8

ADD, acute diarrheal disease; ARR, absolute risk reduction.

1 Mortality rates for infants and children under 5 years were calculated per 1,000 live births, whereas rates for children aged 1 to 4 years were calculated per 1,000 inhabitants in that age group.

2 ARR (the difference in mortality rates between the pre- and post-vaccination periods) is similarly expressed per 1,000.

3 Relative risk reduction is expressed as a percentage.

## Supplementary materials

Supplementary Material 1. Data source summary.

Supplementary Material 2. Population, mortality data, overall and Acute Diarrheal Diseases (ADD) related, rank of ADD in infectious disease mortality, Cause-Specific Mortality Rate (CSMR) and Proportionate mortality (PM) due to ADD among infants, from 1993 to 2021. Means for PM are shown for the three periods analyzed: 1993-1999, 2000-2009 and 2010-2021.

Supplementary Material 3. Population count, mortality data, overall and Acute Diarrheal Diseases (ADD) related, rank of ADD in infectious disease mortality, Cause-Specific Mortality Rate (CSMR) and Proportionate mortality (PM) due to ADD among children aged 1 to 4 years, from 1993 to 2021. Means for PM are shown for the three periods analyzed: 1993-1999, 2000-2009 and 2010-2021.

Supplementary Material 4. Population count, mortality data, overall and Acute Diarrheal Diseases (ADD) related, rank of ADD in infectious disease mortality, Cause-Specific Mortality Rate (CSMR) and Proportionate mortality (PM) due to ADD among children under 5 years old, from 1993 to 2021. Means for PM are shown for the three periods analyzed: 1993-1999, 2000-2009 and 2010-2021.

Supplementary Material 5. Difference among means of proportionate mortality due to ADD between periods (1 vs. 2, 1 vs. 3 and 2 vs. 3) for every population studied.

Supplementary Material 6. Difference among means of cause-specific mortality rates due to ADD between periods (1 vs. 2, 1 vs. 3 and 2 vs. 3) for every population studied. Results are expressed as deaths per 100,000 children of the same age.

Supplementary Material 7. National trends in diarrheal disease, incidence among children under 5 years, infant rotavirus vaccination coverage (Rotarix™, 2 doses), and under-5 mortality rate, Paraguay, 2009–2023.

Supplementary Material 8. Trend analysis using the Joinpoint regression model, based on a log-linear model (ln(y) = βx + e), with years (1992-2022) as independent variable and propionate mortality (PM, %) due to ADD in the pediatric Paraguayan population as the dependent variable: **a)** up to 12 months (infants), **b)** 1–4 years old (1 to 4 years), and **c)** under 5 years old (<5 years). Asterisks indicate the final selected model and the significance level, set at 0.05. Annual percent change (APC) is displayed in the box.
