# Supplementary material for: Childhood mortality from acute diarrheal disease in Paraguay and vaccination impact: a 31-year ecological study
Source: Epidemiol Health. 2026 Feb 20;48:e2026010. doi: 10.4178/epih.e2026010 (PMC13219976; doi:10.4178/epih.e2026010)
Supplement: Supplementary Material 1. — Data source summary. [file epih-48-e2026010-Supplementary-1.docx]

**Supplementary Materials**

**Supplementary Material 1:** Data source summary.

| N | Data source | Organization | Information extracted | Period covered (years) | Data update (dd/mm/yyyy) | Date accessed (dd/mm/yyyy) | References |
| --- | --- | --- | --- | --- | --- | --- | --- |
| 1 | Compulsory Notifiable Disease Dashboard (*Enfermedades de Notificación Obligatoria, ENO*) | General Directorate of Health Surveillance (*Dirección General de Vigilancia Sanitaria, DGVS*) Ministry of Public Health and Social Welfare (*Ministerio de Salud Pública y Bienestar Social, MSPyBS)* (Resolution S,G, No. 190/2013) | Number of reported Acute Diarrheic Disease (ADD) cases, children under 5 years | 2011,  2013 – 2023  (12 years) | 18/07/2024 | 20/07/2024 | (1) |
| 2 | Weekly Epidemiological Bulletins | *DGVS, MSPyBS* (Resolution S,G, No. 0057/2023) | Mortality data, rates due to ADD. | 2006 – 2021 (15 years) | 06/07/2024 | 20/07/2024 | (2) |
| 3 | Historical Series of Mortality Indicators (*Series Históricas de Indicadores de Mortalidad, INDIMOR*) | General Directorate of Strategic Health Information (*Dirección General de Información Estratégica en Salud, DIGIES*) | Population by age-group. Mortality data general and due to ADD, stratified by age group and epidemiological region | 1997 – 2021 (25 years) | 31/12/2021 | 22/06/2024 | (3) |
| 4 | Population Division. World Population Prospects 2022. | United Nations Department of Economic and Social Affairs (UN DESA) | Population recount. Under-five mortality rate | 1993– 1996 (4 years) | 15/11/2022. | 22/06/2024 | (4) |
| 5 | Multiple Indicator Cluster Surveys (MICS) | United Nations Children’s Fund (UNICEF) | Under-five mortality rate | 1993 - 2021 (29 year) | 31/12/2022 | 20/07/2024 | (5) |
| 6 | Vaccination coverage bulletins for children under 5 years old | National Program for Vaccine-Preventable Diseases and Expanded Program on Immunization (*Programa Ampliado de Inmunizaciones, PAI*), *MSPyBS*. | Vaccination against rotavirus, coverage. | 2010 – 2023 (14 years) | 31/12/2023 | 03/08/2024 | (6) |

**References:**

1. Ministerio de Salud Pública y Bienestar Social, Dirección General de Vigilancia de la Salud. Tablas Semanales de Enfermedades de Notificación Obligatoria. [Internet]. Dirección General de Vigilancia de la Salud.: Vigilancia de Eventos de Notificación Obligatoria y Calidad del Dato.; 2024 [cited 2024 Jul 20]. Available from: https://sistemasdgvs.mspbs.gov.py/sistemas/dashboardeno/detalle/dia

2. Ministerio de Salud Pública y Bienestar Social, Dirección General de Vigilancia de la Salud. Sistema Nacional de Vigilancia Epidemiológica del Paraguay. Boletín Epidemiológico Semanal. [Internet]. 2024 [cited 2024 Jul 20]. Available from: https://dgvs.mspbs.gov.py/boletin-epidemiologico-semanal/

3. Ministerio de Salud Pública y Bienestar Social, Dirección General de Información Estratégica en Salud-DIGIES. Series Históricas de Indicadores de Mortalidad (INDIMOR). [Internet]. DIGIES; [cited 2025 Sep 3]. Available from: https://digies.mspbs.gov.py/indimor/

4. United Nations Department of Economic and Social Affairs. World population prospects 2022: summary of results. New York: United Nations; 2022.

5. UNICEF. Multiple Indicator Cluster Surveys-MICS. Country Profile: Paraguay. [Internet]. UNICEF Data: Multiple Indicator Cluster Surveys-MICS; [cited 2024 Jul 20]. Available from: https://data.unicef.org/country/pry/

6. Programa Ampliado de Inmunizaciones-PAI. Boletines de cobertura de vacunación de menores de 5 años. [Internet]. [cited 2024 Aug 3]. Available from: https://pai.mspbs.gov.py/boletines-de-coberturas-de-vacunacion-de-menor-de-5-anos/
