# Supplementary material for: Childhood mortality from acute diarrheal disease in Paraguay and vaccination impact: a 31-year ecological study
Source: Epidemiol Health. 2026 Feb 20;48:e2026010. doi: 10.4178/epih.e2026010 (PMC13219976; doi:10.4178/epih.e2026010)
Supplement: Supplementary Material 2. — Population, mortality data, overall and Acute Diarrheal Diseases (ADD) related, rank of ADD in infectious disease mortality, Cause-Specific Mortality Rate (CSMR) and Proportionate mortality (PM) due to ADD among infants, from 1993 to 2021. Means for PM are shown for the three periods analyzed: 1993-1999, 2000-2009 and 2010-2021. [file epih-48-e2026010-Supplementary-2.docx]

**Supplementary Material 2:** Population, mortality data, overall and Acute Diarrheal Diseases (ADD) related, rank of ADD in infectious disease mortality, Cause-Specific Mortality Rate (CSMR) and Proportionate mortality (PM) due to ADD among infants, from 1993 to 2021. Means for PM are shown for the three periods analyzed: 1993-1999, 2000-2009 and 2010-2021.

| **Age range** | **Infant** | | | | | | | |
| --- | --- | --- | --- | --- | --- | --- | --- | --- |
| **Years** | **Population - live births (No.)** | **Mortality - All causes (No.)** | **Mortality - ADD (No.)** | **Mortality rates – ADD**  **(/ 1,000 live births)** | **ADD rank in infectious disease mortality** | **CSMR**  **(/ 100,000 infants)** | **PM due to ADD (%)** | **PM - Mean per period** |
| **1993** | 154200 | 1677 | 233 | 3.0 | 3º | 151 | 13.89 | 11.25 |
| **1994** | 156100 | 1460 | 265 | 3.3 | 1º | 170 | 18.15 |  |
| **1995** | 158000 | 1383 | 187 | 2.4 | 3º | 118 | 13.52 |  |
| **1996** | 160000 | 1670 | 178 | 2.0 | 3º | 111 | 10.66 |  |
| **1997** | 162000 | 1569 | 170 | 1.9 | 3º | 105 | 10.83 |  |
| **1998** | 164000 | 1522 | 177 | 2.0 | 3º | 108 | 11.63 |  |
| **1999** | 166000 | 1582 | 167 | 1.9 | 3º | 101 | 10.56 |  |
| **2000** | 168000 | 1572 | 165 | 2.0 | 2º | 98 | 10.50 | 5.13 |
| **2001** | 150407 | 1535 | 117 | 1.0 | 3º | 78 | 7.62 |  |
| **2002** | 150844 | 1653 | 114 | 1.3 | 3º | 76 | 6.90 |  |
| **2003** | 151281 | 1589 | 94 | 1.1 | 3º | 62 | 5.92 |  |
| **2004** | 151718 | 1636 | 78 | 1.0 | 3º | 51 | 4.77 |  |
| **2005** | 152155 | 1776 | 103 | 1.0 | 2º | 68 | 5.80 |  |
| **2006** | 152336 | 1771 | 68 | 1.0 | 3º | 45 | 3.84 |  |
| **2007** | 152517 | 1561 | 43 | 0.4 | 3º | 28 | 2.75 |  |
| **2008** | 152698 | 1624 | 58 | 1.0 | 2º | 38 | 3.57 |  |
| **2009** | 152879 | 1538 | 39 | 0.38 | 3º | 26 | 2.54 |  |
| **2010** | 153060 | 1609 | 42 | 0.42 | 3º | 27 | 2.61 | 1.35 |
| **2011** | 153012 | 1578 | 29 | 0.3 | 3º | 19 | 1.84 |  |
| **2012** | 152964 | 1567 | 23 | 0.2 | 3º | 15 | 1.47 |  |
| **2013** | 152916 | 1550 | 12 | 0.1 | 3º | 8 | 0.77 |  |
| **2014** | 152868 | 1613 | 23 | 0.2 | 3º | 15 | 1.43 |  |
| **2015** | 144445 | 1631 | 18 | 0.2 | 3º | 12 | 1.10 |  |
| **2016** | 144593 | 1505 | 17 | 0.2 | 3º | 12 | 1.13 |  |
| **2017** | 144695 | 1444 | 17 | 0.1 | 3º | 12 | 1.18 |  |
| **2018** | 144794 | 1458 | 19 | 0.2 | 3º | 13 | 1.30 |  |
| **2019** | 144877 | 1293 | 15 | 0.1 | 3º | 10 | 1.16 |  |
| **2020** | 144940 | 1241 | 16 | 0.2 | 2º | 11 | 1.29 |  |
| **2021** | 144997 | 1395 | 14 | 0.1 | 3º | 10 | 1 |  |
| **Total** | **4433296** | **45002** | **2501** | **0.6** | **3º** | **56** | **5.56** |  |
