# Supplementary material for: Childhood mortality from acute diarrheal disease in Paraguay and vaccination impact: a 31-year ecological study
Source: Epidemiol Health. 2026 Feb 20;48:e2026010. doi: 10.4178/epih.e2026010 (PMC13219976; doi:10.4178/epih.e2026010)
Supplement: Supplementary Material 3. — Population count, mortality data, overall and Acute Diarrheal Diseases (ADD) related, rank of ADD in infectious disease mortality, Cause-Specific Mortality Rate (CSMR) and Proportionate mortality (PM) due to ADD among children aged 1 to 4 years, from 1993 to 2021. Means for PM are shown for the three periods analyzed: 1993-1999, 2000-2009 and 2010-2021. [file epih-48-e2026010-Supplementary-3.docx]

**Supplementary Material 3:** Population count, mortality data, overall and Acute Diarrheal Diseases (ADD) related, rank of ADD in infectious disease mortality, Cause-Specific Mortality Rate (CSMR) and Proportionate mortality (PM) due to ADD among children aged 1 to 4 years, from 1993 to 2021. Means for PM are shown for the three periods analyzed: 1993-1999, 2000-2009 and 2010-2021.

| **Age range** | **1 to 4 years old** | | | | | | | |
| --- | --- | --- | --- | --- | --- | --- | --- | --- |
| **Years** | **Population - aged 1 to 4 years (No.)** | **Mortality - All causes (No.)** | **Mortality - ADD (No.)** | **Mortality rates – ADD**  **(/ 100,000 aged 1 to 4 years)** | **ADD rank in infectious disease mortality** | **CSMR**  **(/ 100,000 aged 1 to 4 years)** | **PM due to ADD (%)** | **PM - Mean per period** |
| **1993** | 552923 | 628 | 140 | 25.32 | 2º | 25 | 22.29 | 18.32 |
| **1994** | 573199 | 435 | 148 | 25.82 | 2º | 26 | 34.02 |  |
| **1995** | 581738 | 373 | 79 | 13.58 | 2º | 14 | 21.18 |  |
| **1996** | 587838 | 438 | 87 | 14.8 | 2º | 15 | 19.86 |  |
| **1997** | 594126 | 393 | 89 | 14.98 | 2º | 15 | 22.65 |  |
| **1998** | 601109 | 396 | 75 | 12.48 | 2º | 12 | 18.94 |  |
| **1999** | 607389 | 391 | 67 | 11 | 2º | 11 | 17.14 |  |
| **2000** | 615787 | 396 | 71 | 9.18 | 2º | 12 | 17.93 | 12.15 |
| **2001** | 625000 | 381 | 65 | 10.4 | 2º | 10 | 17.06 |  |
| **2002** | 638491 | 376 | 65 | 10.2 | 2º | 10 | 17.29 |  |
| **2003** | 591350 | 346 | 42 | 6.7 | 2º | 7 | 12.14 |  |
| **2004** | 578195 | 386 | 71 | 12.3 | 2º | 12 | 18.29 |  |
| **2005** | 580681 | 380 | 46 | 7.9 | 2º | 8 | 12.11 |  |
| **2006** | 582830 | 341 | 34 | 5.8 | 2º | 6 | 9.97 |  |
| **2007** | 585101 | 277 | 22 | 3.8 | 2º | 4 | 7.94 |  |
| **2008** | 586306 | 290 | 28 | 4.8 | 2º | 5 | 9.66 |  |
| **2009** | 588348 | 298 | 36 | 6.1 | 2º | 6 | 12.08 |  |
| **2010** | 589681 | 272 | 30 | 5.1 | 2º | 5 | 11.03 | 4.72 |
| **2011** | 590818 | 241 | 8 | 1.4 | 2º | 1 | 3.32 |  |
| **2012** | 591746 | 242 | 3 | 0.5 | 4º | 1 | 1.24 |  |
| **2013** | 592477 | 243 | 14 | 2.4 | 2º | 2 | 5.76 |  |
| **2014** | 593025 | 235 | 16 | 2.7 | 2º | 3 | 6.81 |  |
| **2015** | 559279 | 242 | 9 | 1.6 | 2º | 2 | 3.72 |  |
| **2016** | 560545 | 253 | 13 | 2.3 | 2º | 2 | 5.14 |  |
| **2017** | 561556 | 209 | 8 | 1.4 | 2º | 1 | 3.83 |  |
| **2018** | 562537 | 238 | 13 | 2.3 | 2º | 2 | 5.46 |  |
| **2019** | 563392 | 267 | 16 | 2.8 | 2º | 3 | 5.99 |  |
| **2020** | 564181 | 204 | 7 | 1.2 | 2º | 1 | 3.43 |  |
| **2021** | 564843 | 219 | 5 | 0.9 | 3º | 1 | 2 |  |
| **Total** | **16964490** | **9390** | **1307** | **7.7** | **2º** | **8** | **13.92** |  |
