# Supplementary material for: Childhood mortality from acute diarrheal disease in Paraguay and vaccination impact: a 31-year ecological study
Source: Epidemiol Health. 2026 Feb 20;48:e2026010. doi: 10.4178/epih.e2026010 (PMC13219976; doi:10.4178/epih.e2026010)
Supplement: Supplementary Material 4. — Population count, mortality data, overall and Acute Diarrheal Diseases (ADD) related, rank of ADD in infectious disease mortality, Cause-Specific Mortality Rate (CSMR) and Proportionate mortality (PM) due to ADD among children under 5 years old, from 1993 to 2021. Means for PM are shown for the three periods analyzed: 1993-1999, 2000-2009 and 2010-2021. [file epih-48-e2026010-Supplementary-4.docx]

**Supplementary Material 4:** Population count, mortality data, overall and Acute Diarrheal Diseases (ADD) related, rank of ADD in infectious disease mortality, Cause-Specific Mortality Rate (CSMR) and Proportionate mortality (PM) due to ADD among children under 5 years old, from 1993 to 2021. Means for PM are shown for the three periods analyzed: 1993-1999, 2000-2009 and 2010-2021.

| **Age range** | **Under 5 years old** | | | | | | | |
| --- | --- | --- | --- | --- | --- | --- | --- | --- |
| **Years** | **Population - aged < 5 years (No.)** | **Mortality - All causes (No.)** | **Mortality - ADD (No.)** | **Mortality rates – ADD**  **(/ 1,000 live births)**^1^ | **ADD rank in infectious disease mortality** | **CSMR**  **(/ 100,000 < 5 years)** | **PM due to ADD (%)** | **PM - Mean per period** |
| **1993** | 669660 | 2305 | 373 | 4.78 | Nd^2^ | 56 | 16.18 | 12.86 |
| **1994** | 675907 | 1895 | 413 | 5.19 | Nd | 61 | 21.79 |  |
| **1995** | 680922 | 1756 | 266 | 3.34 | Nd | 39 | 15.15 |  |
| **1996** | 684411 | 2108 | 265 | 2.99 | Nd | 39 | 12.57 |  |
| **1997** | 725096 | 1962 | 259 | 2.93 | 3º | 36 | 13.20 |  |
| **1998** | 756550 | 1927 | 243 | 2.8 | 3º | 32 | 12.61 |  |
| **1999** | 764886 | 1971 | 235 | 2.6 | 3º | 31 | 11.92 |  |
| **2000** | 773714 | 1968 | 236 | 3 | 3º | 31 | 11.99 | 6.43 |
| **2001** | 685427 | 1916 | 182 | 2 | 3º | 27 | 9.50 |  |
| **2002** | 796084 | 2029 | 179 | 2 | 3º | 22 | 8.82 |  |
| **2003** | 741778 | 1935 | 136 | 1.6 | 3º | 18 | 7.03 |  |
| **2004** | 725592 | 2022 | 149 | 1 | 3º | 21 | 7.37 |  |
| **2005** | 728551 | 2156 | 149 | 1 | 2º | 20 | 6.91 |  |
| **2006** | 731068 | 2112 | 102 | 1 | 3º | 14 | 4.83 |  |
| **2007** | 733206 | 1839 | 64 | 1 | 3º | 9 | 3.48 |  |
| **2008** | 735042 | 1917 | 83 | 1 | 2º | 11 | 4.33 |  |
| **2009** | 736653 | 1836 | 75 | 1 | 3º | 10 | 4.08 |  |
| **2010** | 738114 | 1881 | 72 | 1 | 3º | 10 | 3.83 | 1.82 |
| **2011** | 739448 | 1819 | 37 | 0.3 | 3º | 5 | 2.03 |  |
| **2012** | 740605 | 1809 | 26 | 0.2 | 3º | 4 | 1.44 |  |
| **2013** | 741550 | 1793 | 26 | 0.2 | 3º | 4 | 1.45 |  |
| **2014** | 742249 | 1848 | 39 | 0.3 | 3º | 5 | 2.11 |  |
| **2015** | 700638 | 1873 | 27 | 0.2 | 3º | 4 | 1.44 |  |
| **2016** | 702082 | 1758 | 30 | 0.3 | 3º | 4 | 1.71 |  |
| **2017** | 703313 | 1654 | 24 | 0.2 | 3º | 3 | 1.45 |  |
| **2018** | 704378 | 1697 | 31 | 0.3 | 3º | 4 | 1.83 |  |
| **2019** | 705443 | 1560 | 31 | 0.3 | 3º | 4 | 1.99 |  |
| **2020** | 706285 | 1445 | 23 | 0.2 | 3º | 3 | 1.59 |  |
| **2021** | 707118 | 1615 | 18 | 0.2 | 3º | 3 | 1 |  |
| **Total** | **20975770** | **54406** | **3793** | **0.9** | **3º** | **18** | **6.97** |  |
| ***1.***  *As reported by INDIMOR*  ***2.*** *Nd: No data available* | | | | | | | | |
