# Supplementary material for: Childhood mortality from acute diarrheal disease in Paraguay and vaccination impact: a 31-year ecological study
Source: Epidemiol Health. 2026 Feb 20;48:e2026010. doi: 10.4178/epih.e2026010 (PMC13219976; doi:10.4178/epih.e2026010)
Supplement: Supplementary Material 5. — Difference among means of proportionate mortality due to ADD between periods (1 vs. 2, 1 vs. 3 and 2 vs. 3) for every population studied. [file epih-48-e2026010-Supplementary-5.docx]

**Supplementary Material 5:** Difference among means of proportionate mortality due to ADD between periods (1 vs. 2, 1 vs. 3 and 2 vs. 3) for every population studied.

| **Population** | **Period** | **Difference between proportions (%)** | **Z-score** | **p-value** |
| --- | --- | --- | --- | --- |
| Infants | 1 vs. 2 | 6.10 | 7.10 | < 0.0001 |
|  | 1 vs. 3 | 9.90 | 10.65 | < 0.0001 |
|  | 2 vs. 3 | 3.80 | 5.47 | < 0.0001 |
| Children aged 1 to 4 years | 1 vs. 2 | 6.17 | 5.91 | < 0.0001 |
|  | 1 vs. 3 | 13.60 | 11.57 | < 0.0001 |
|  | 2 vs. 3 | 7.43 | 7.41 | < 0.0001 |
| Under 5 years | 1 vs. 2 | 6.43 | 5.17 | < 0.0001 |
|  | 1 vs. 3 | 11.04 | 9.41 | < 0.0001 |
|  | 2 vs. 3 | 4.61 | 5.13 | < 0.0001 |
