# Supplementary material for: Childhood mortality from acute diarrheal disease in Paraguay and vaccination impact: a 31-year ecological study
Source: Epidemiol Health. 2026 Feb 20;48:e2026010. doi: 10.4178/epih.e2026010 (PMC13219976; doi:10.4178/epih.e2026010)
Supplement: Supplementary Material 6. — Difference among means of cause-specific mortality rates due to ADD between periods (1 vs. 2, 1 vs. 3 and 2 vs. 3) for every population studied. Results are expressed as deaths per 100,000 children of the same age. [file epih-48-e2026010-Supplementary-6.docx]

**Supplementary Material 6:** Difference among means of cause-specific mortality rates due to ADD between periods (1 vs. 2, 1 vs. 3 and 2 vs. 3) for every population studied. Results are expressed as deaths per 100,000 children of the same age.

| **Population** | **CSMR (means)** | **Periods** | **Difference between means** |
| --- | --- | --- | --- |
| **Infants (deaths per 100,000 infants)** | | | |
| Period 1 | 123 | 1 vs. 2 | 66 |
| Period 2 | 57 | 1 vs. 3 | 109 |
| Period 3 | 14 | 2 vs. 3 | 43 |
| **Children aged 1 to 4 years (deaths per 100,000 children 1 to 4 years)** | | | |
| Period 1  Period 2  Period 3 | 17 | 1 vs. 2 | 9 |
|  | 8 | 1 vs. 3 | 15 |
|  | 2 | 2 vs. 3 | 6 |
| **Children under 5 years (deaths per 100,000 children under 5 years)** | | | |
| Period 1  Period 2  Period 3 | 42 | 1 vs. 2 | 24 |
|  | 18 | 1 vs. 3 | 38 |
|  | 4 | 2 vs. 3 | 14 |
