# Supplementary material for: Childhood mortality from acute diarrheal disease in Paraguay and vaccination impact: a 31-year ecological study
Source: Epidemiol Health. 2026 Feb 20;48:e2026010. doi: 10.4178/epih.e2026010 (PMC13219976; doi:10.4178/epih.e2026010)
Supplement: Supplementary Material 7. — National trends in diarrheal disease, incidence among children under 5 years, infant rotavirus vaccination coverage (Rotarix™, 2 doses), and under-5 mortality rate, Paraguay, 2009–2023. [file epih-48-e2026010-Supplementary-7.docx]

**Supplementary Material 7:** National trends in diarrheal disease, incidence among children under 5 years, infant rotavirus vaccination coverage (Rotarix™, 2 doses), and under-5 mortality rate, Paraguay, 2009–2023.

| **Year** | **ADD cases (<5 years) (/ 1,000 <5 years)** | **Vaccination against rotavirus (Infants, %)** | **Mortality rate (<5 years) (/ 1,000 live births)** |
| --- | --- | --- | --- |
| **2009** | Nd^1^ | Nd | 1 |
| **2010** | Nd | 55 | 1 |
| **2011** | 68.3 | 72 | 0.3 |
| **2012** | Nd | 71 | 0.2 |
| **2013** | 77.9 | 70 | 0.2 |
| **2014** | 91.7 | 72 | 0.3 |
| **2015** | 102.6 | 79 | 0.2 |
| **2016** | 111.9 | 79 | 0.3 |
| **2017** | 120.8 | 81 | 0.2 |
| **2018** | 134.1 | 79 | 0.3 |
| **2019** | 148.9 | 74 | 0.3 |
| **2020** | 55.5 | 68 | 0.2 |
| **2021** | 68.7 | 56 | 0.2 |
| **2022** | 89.3 | 65 | Nd |
| **2023** | 83.7 | 67 | Nd |
| ^1^ Nd= no data available | | | |
