# Supplementary material for: Childhood mortality from acute diarrheal disease in Paraguay and vaccination impact: a 31-year ecological study
Source: Epidemiol Health. 2026 Feb 20;48:e2026010. doi: 10.4178/epih.e2026010 (PMC13219976; doi:10.4178/epih.e2026010)
Supplement: Supplementary Material 8. — Trend analysis using the Joinpoint regression model, based on a log-linear model (ln(y) = βx + e), with years (1992-2022) as independent variable and propionate mortality (PM, %) due to ADD in the pediatric Paraguayan population as the dependent variable: a) up to 12 months (infants), b) 1–4 years old (1 to 4 years), and c) under 5 years old (<5 years). Asterisks indicate the final selected model and the significance level, set at 0.05. Annual percent change (APC) is displayed in the box. [file epih-48-e2026010-Supplementary-8.docx]

**a)**

**
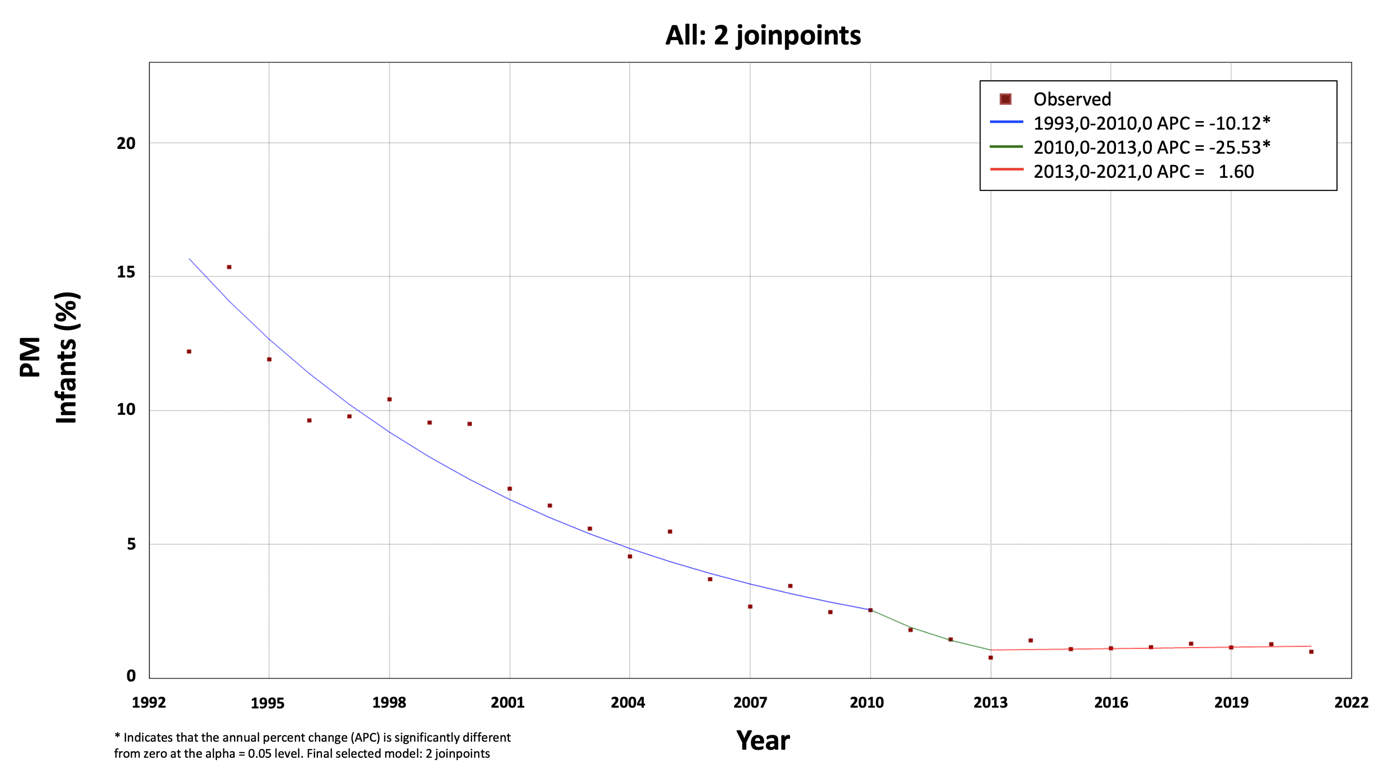
**

**b)**


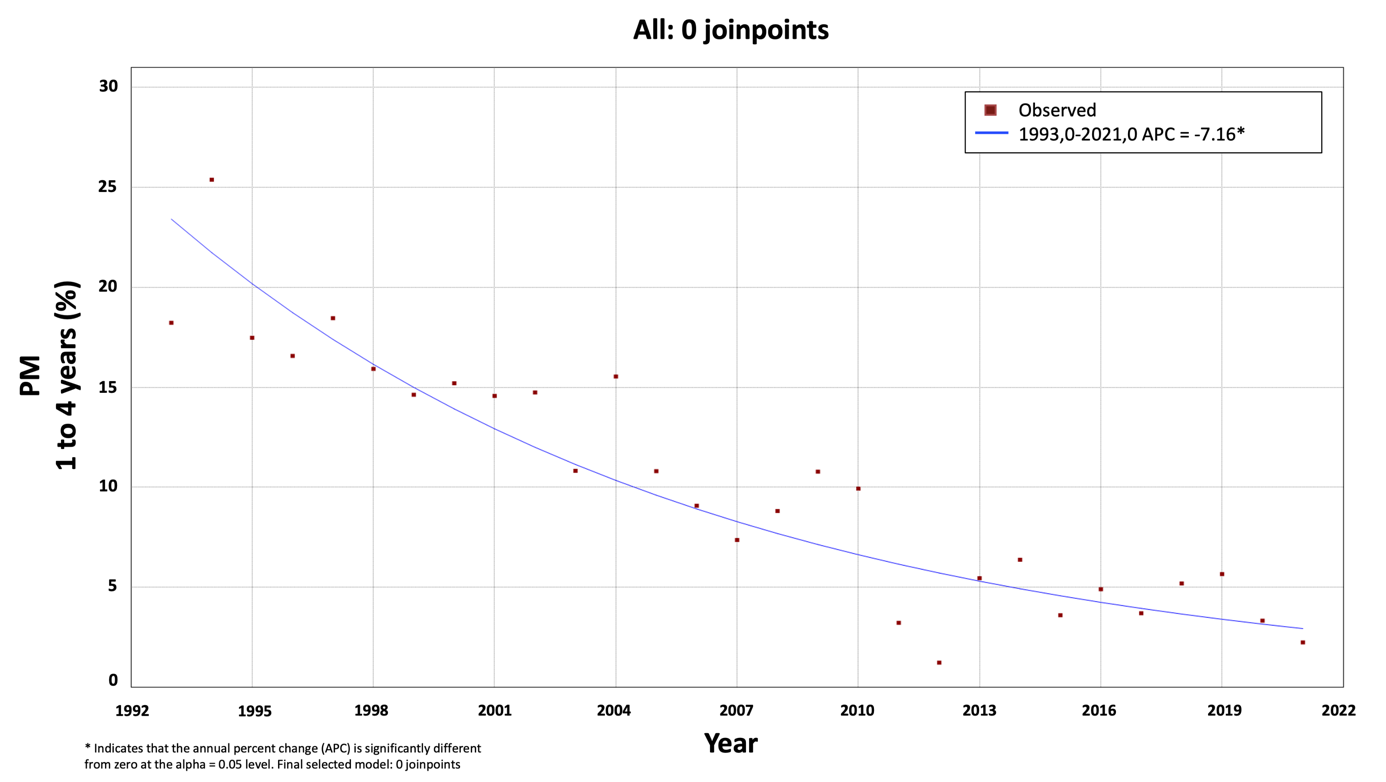


**(Continues)**

**(Continued)**

**c)**

**
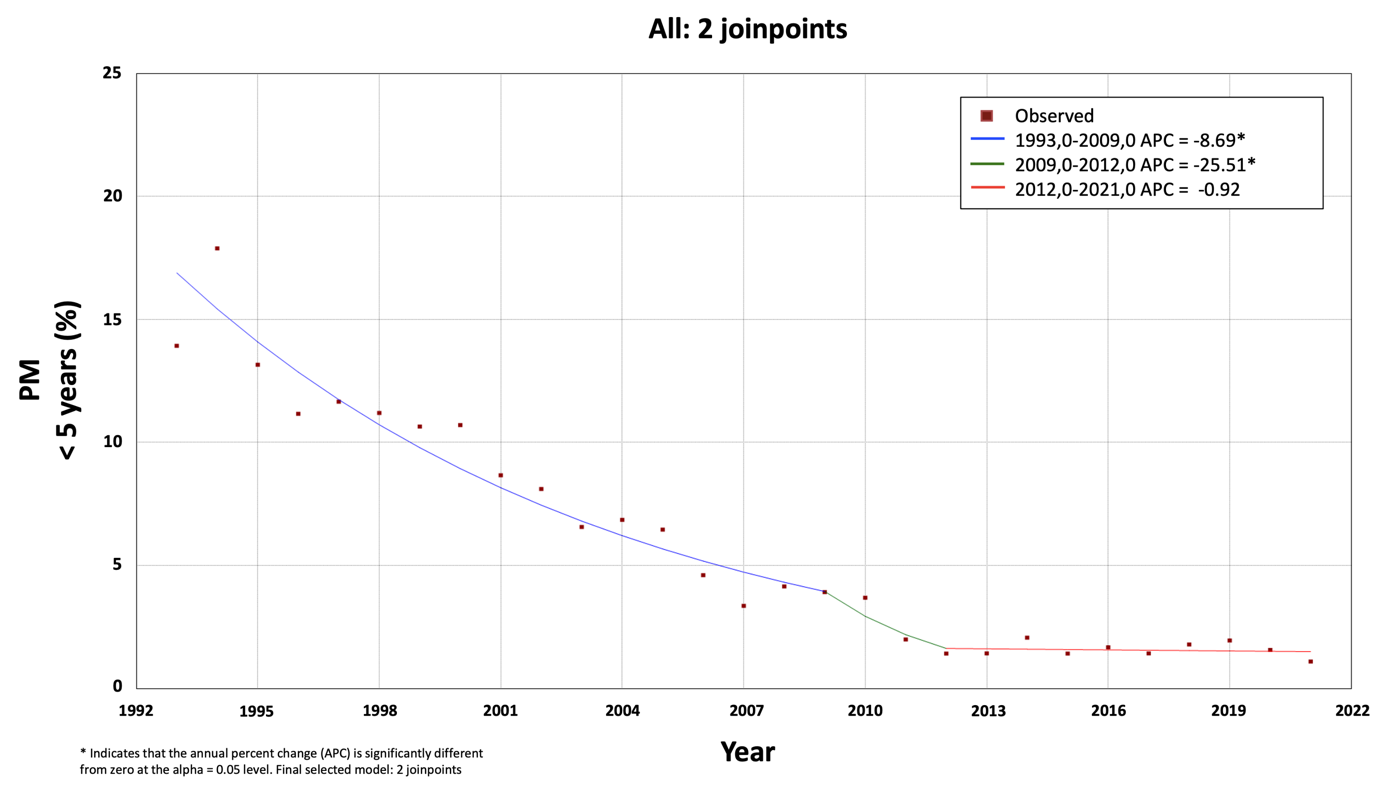
**

**Supplementary Material 8.** Trend analysis using the Joinpoint regression model, based on a log-linear model (ln(y) = βx + e), with years (1992-2022) as independent variable and propionate mortality (PM, %) due to ADD in the pediatric Paraguayan population as the dependent variable: **a)** up to 12 months (infants), **b)** 1–4 years old (1 to 4 years), and **c)** under 5 years old (<5 years). Asterisks indicate the final selected model and the significance level, set at 0.05. Annual percent change (APC) is displayed in the box.
